# Development of an Expression Vector to Overexpress or Downregulate Genes in *Curvularia protuberata*

**DOI:** 10.3390/jof4020054

**Published:** 2018-05-05

**Authors:** Chengke Liu, Blake Cleckler, Mustafa Morsy

**Affiliations:** The Department of Biological and Environmental Sciences, University of West Alabama, Livingston, AL 35470, USA; cliu@uwa.edu (C.L.); bcleckler@uwa.edu (B.C.)

**Keywords:** *ToxA* promoter, zeocin resistance, *Curvularia protuberata*, *Curvularia* thermotolerance virus, overexpression, downregulation

## Abstract

*Curvularia protuberata*, an endophytic fungus in the Ascomycota, provides plants with thermotolerance only when it carries a mycovirus known as *Curvularia* thermotolerance virus (CThTV), and forms a three-way symbiotic relationship among these organisms. Under heat stress, several genes are expressed differently between virus-free *C. protuberata* (VF) and *C. protuberata* carrying CThTV (AN). We developed an expression vector, pM2Z-fun, carrying a zeocin resistance gene driven by the *ToxA* promoter, to study gene functions in *C. protuberata* to better understand this three-way symbiosis. Using this new 3.7-kb vector, five genes that are differentially expressed in *C. protuberata*—including genes involved in the trehalose, melanin, and catalase biosynthesis pathways—were successfully overexpressed or downregulated in VF or AN *C. protuberata* strains, respectively. The VF overexpression lines showed higher metabolite and enzyme activity than in the control VF strain. Furthermore, downregulation of expression of the same genes in the AN strain resulted in lower metabolite and enzyme activity than in the control AN strain. The newly generated expression vector, pM2Z-fun, has been successfully used to express target genes in *C. protuberata* and will be useful in further functional expression studies in other Ascomycota fungi.

## 1. Introduction

The endophytic fungus *Curvularia protuberata* carrying the mycovirus *Curvularia* thermotolerance virus (CThTV) can participate in a three-way symbiosis with plants that leads to extreme thermotolerance [1]. *C. protuberata* confers plant thermotolerance only when the CThTV is present, but neither the virus-free fungus (VF) nor plant can survive extremely high soil temperature (65 °C) independently. The *C. protuberata* AN strain, which was produced by hyphal anastomosis of VF and wild-type *C. protuberata*, regains the ability to confer thermotolerance [1]. This virus–fungus–plant three-way symbiosis has been discovered in monocot (*Dichanthelium lanuginosum*) and was confirmed in dicot (*Solanum lycopersicon*) plants also, which suggests a conserved thermotolerance mechanism [1,2]. In order to make the best use of this three-way symbiosis to improve crop thermotolerance, it is necessary to understand the molecular mechanisms that govern this system. Therefore, in order to study the *C. protuberata* gene functions and their roles in acquired thermotolerance, we constructed an expression vector, pM2Z-fun, using the *ToxA* gene promotor and zeocin resistance gene as a selective marker.

The promoter of the *ToxA* gene, a necrosis-inducing host-selective toxin gene from *Pyrenophora tritici-repentis* [3], was used to drive expression in the vector pCT74 [4]. This vector has been used to express a reporter gene encoding green fluorescent protein, which causes bright cytoplasmic fluorescence in eight ascomycete fungal genera [4]. The *ToxA* promoter has also been used successfully to drive expression of other fluorescent proteins in several related fungi [3,4,5].

We are using a vector that carries the 370-bp *Sh ble* gene, which confers resistance to zeocin [6,7], an antibiotic that causes cell death by cleaving DNA that has been widely used as selective marker for transformation of fungi, algae, and mammalian cells [8,9,10,11]. In some cases, selection using zeocin results in higher transformation efficiencies than selection using other antibiotics [12,13].

Because several *C. protuberata* genes are differently expressed between AN and VF strains under heat stress, we hypothesize that these genes are involved in the thermotolerance mechanism that results from this three-way symbiosis [2]. Five of these genes were chosen to test the new expression vector. These target genes included genes in the melanin synthesis pathway: 1,3,6,8-tetrahydroxynaphthalene reductase (*T4HN*) and scytalone dehydratase (*SCD*); genes in the trehalose synthesis pathway: trehalose-6-phosphate synthase (*TPS1*) and trehalose-6-phosphate phosphatase (*TPS2*); and a catalase/peroxidase gene (*CAT*) [2].

Melanin is a pigment formed by polymerization of phenolic compounds that protects organisms from ultraviolet radiation and environmental stressors [14,15,16,17]. The two dominant types of melanin in fungi are dihydroxynaphthalene (DHN)-melanin and dihydroxyphenylalanine (DOPA)-melanin [18]. Expression of the DHN-melanin biosynthesis genes in *Metarhizium anisopliae* enhances stress tolerance and virulence [19]. *T4HN* and *SCD* are other key genes involved in the DHN-melanin biosynthesis pathway [20,21]. Interestingly, expression of both *T4HN* and *SCD* transcripts in *Bipolaris oryzae* is enhanced by near-ultraviolet irradiation [22].

Trehalose, a non-reducing disaccharide present in bacteria, fungi, plants, and invertebrates [23,24], serves as a carbohydrate storage molecule, developmental regulator, and abiotic stress protectant [25,26,27,28]. Trehalose is synthesized in two steps: first, trehalose phosphate synthase (*TPS1*) catalyzes the synthesis of trehalose-6-phosphase from gluose-6-phosphate and uridine diphosphate-glucose; second, trehalose-6-phosphate phosphatase (*TPS2*) catalyzes the dephosphorylation of trehalose-6-phosphate to trehalose [24,29]. A mutation in the *TPS1* gene of *Botrytis cinerea* prevents trehalose synthesis and leads to increased heat sensitivity of the mutant compared to the wild type [30].

Hydrogen peroxide (H_2_O_2_) is a reactive oxygen species that can cause severe cellular damage. It can be degraded and catalyzed into water (H_2_O) and oxygen (O_2_) by the enzyme catalase/peroxidase, which is present in all aerobic organisms [31,32]. Catalase is also used to protect cells from oxidative damages associated with a variety of stresses [33,34,35,36]. In addition, overexpression of catalase in fungi improves their spore germination and mycelial growth rate [36,37].

In this article, we demonstrate the differences in metabolite and enzyme activity between overexpressed and downregulated targeted genes in *C. protuberata* VF and AN stains, respectively, using the newly developed expression vector pM2Z-fun, to further the understanding of the molecular mechanisms that drive this plant, fungus, and virus three-way symbiotic relationship.

## 2. Materials and Methods

### 2.1. Fungal Culture

All fungal strains of *C. protuberata* (VF, AN, and their transformants) were cultured on 0.1× potato dextrose agar (PDA) plates (pH 5.8) or in 1× potato dextrose (PD) liquid medium (pH 5.8) supplemented with ampicillin (100 µg/mL), kanamycin (50 µg/mL), and streptomycin (100 µg/mL). Different concentrations (described below) of zeocin were added for selection of fungal transformants.

### 2.2. Vector Construction

To generate the fungal expression vector pM2Z-fun, a multiple cloning site (MCS) cassette containing *Eco*RI, KpnI, PstI, *Bam*HI, *Spe*I, *Hin*dIII, and *Xba*I, terminator NOS (Genebank ID: KY031321.1), the *ToxA* promoter (Genebank ID: DQ423483.1), zeocin cassette containing pTEF1 promoter, *Sh ble* gene (Genebank ID: KY793908.1), and terminator CYC1 (Genebank ID: KM035419.1) was synthesized by Invitrogen (Invitrogen, Waltham, MA, USA) and inserted into pMZ vector. The newly synthesized vector was used as the backbone for both overexpression and RNAi vectors for the target genes. The vector described in this paper is available to share by contacting the corresponding author.

### 2.3. Overexpression and RNAi Constructs

Total RNA was extracted from 3 mg of lyophilized AN strain mycelia using a PureLink^®^ RNA Mini Kit (Thermo Fisher Scientific, Waltham, MA, USA). First-strand cDNA was synthesized from 1 µg RNA using Oligo-dT primers and *Moloney murine* leukemia virus reverse transcriptase (Promega, Madison, WI, USA). To clone genes of interest for overexpression, primers containing specific restriction site sequences were designed according to our EST data (Table 1). Each gene of interest was amplified by PCR using Phusion^®^ High-Fidelity DNA polymerase (New England Biolabs, Ipswich, MA, USA), then PCR products purified, cleaved with the appropriate restriction enzyme, and cloned into the pM2Z-fun vector. All clones were sequenced to confirm the presence of expected genes in the correct sequence.

For the RNAi vectors, the sense fragment (A) and anti-sense fragment (B) of each target gene was amplified separately, and then inserted into the pM2Z-fun vector sequentially (primers and restriction enzyme sites are listed in Table 1). For each specific gene, the forward primer of the sense fragment and the reverse primer of the antisense fragment were the same. The 5′ end of the anti-sense fragment was about 100-bp (±10-bp, varied according to specific gene) shorter than the sense fragment to allow hairpin formation. The vectors were digested with *Eco*RI to confirm the insertion.

### 2.4. Protoplast Isolation

Fungal protoplasts were isolated using the method described by Young [38] with modifications. Five-day-old mycelia cultures were harvested for protoplast preparation. One gram of wet mycelia was resuspended in 30 mL of enzyme buffer (1.2 M MgSO_4_, 10 mM K_2_HPO_4_, pH 5.8) containing 1.2% lysing enzyme (Sigma, St. Louis, MO, USA) and shaken at 50 rpm in the dark for 4 h with gradually increased temperatures: 26 °C for 30 min, 30 °C for 30 min, 33 °C for 30 min, 35 °C for 2 h, and finally 37 °C for 30 min. The protoplasts were harvested and washed 3× using STC buffer (1 M sorbitol, 50 mM Tris, pH 5.8, 50 mM CaCl_2_). The protoplasts were resuspended in STC buffer at a final concentration of 1 × 10^8^ cells/mL.

### 2.5. Transformation and Screening

Protoplast transformation was carried out as described by Itoh [39] with modifications. Transformants were selected on HM media (138.5 g mannitol, 1 g casamino acids, 1 g yeast extract, 4 g sucrose and 20 g agar per 1 L) plates containing 50 µg/mL zeocin. The resulting transformants were subsequently maintained on PDA containing 20 µg/mL zeocin.

Potential fungal transformants were screened for the presence of inserted genes by PCR with forward primers for *ToxA* and reverse primers targeting each specific target gene (Table 1).

### 2.6. Semi-Quantitative Reverse Transcription-PCR

Seven-day-old liquid fungus cultures were vacuum filtered and washed with sterile H_2_O. The collected mycelia were then freeze-dried overnight. Total RNA extraction and synthesis of first-strand cDNA were performed as described above. To quantify the expression of specific genes, 1 µL of first-strand cDNA was used with GoTaq (Promega, Madison, WI, USA) and 5× green GoTaq Reaction Buffer in each 20 µL PCR reaction for 25–27 amplification cycles at 57–60 °C for annealing temperature depending on the specific gene (primers are listed in Table 1). The glyceradehyde-3-phosphate dehydrogenase (*GPD*) gene was used as an internal control.

### 2.7. Melanin Extraction and Quantification

Melanin extraction and analysis were performed as described by Fernandes [40] with minor modifications. Briefly, 1 M NaOH was added to 20 mg of freeze-dried mycelia (1 mL/10 mg) and the pigment was extracted by autoclaving at 121 °C for 60 min. Samples were centrifuged and the collected supernatant was used to spectrophotometrically quantify melanin content by absorbance at 405 nm. Three independent samples were analyzed.

### 2.8. Trehalose Assay

Fungal trehalose was extracted from five-day-old liquid cultures as described previously with modifications [41]. Fungal culture was vacuum filtered and washed with distilled H_2_O. Two volumes of distilled H_2_O were added to the washed mycelia and the samples were boiled for 20 min to inactivate enzymes and release soluble sugars. The supernatant was collected by centrifugation at 12,000 rpm for 5 min. Free D-glucose was removed from the supernatant, and then a trehalose assay was performed using a Trehalose Assay Kit (Megazyme, Chicago, IL, USA) following the manufacturer’s recommendations. Three independent samples were analyzed.

### 2.9. Catalase Assay

The analysis of catalase activity was performed as described by Iwase [42] with modifications. Five-day-old liquid fungal culture was homogenized before harvesting. A 30-mg sample of washed fungal mycelia was weighed and added to the bottom of a 20 mL glass tube. Five milliliters of 3% H_2_O_2_ containing 1% Triton-100 was slowly added into the tube. The foam formed by the reaction between catalase and H_2_O_2_ was measured after 5 min. Catalase activity was determined as the depth of the foam measured in centimeters.

## 3. Results

### 3.1. Vector Construction

**Figure 1:**
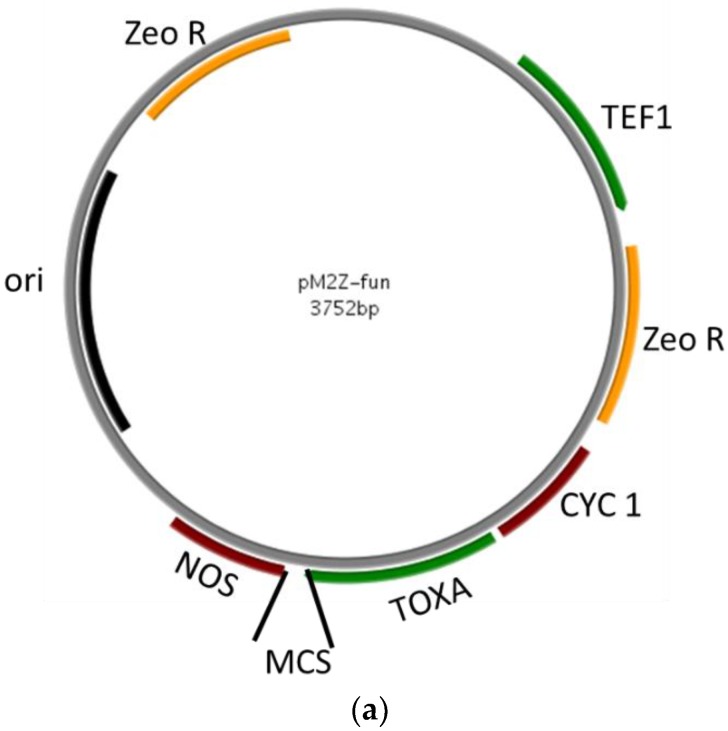

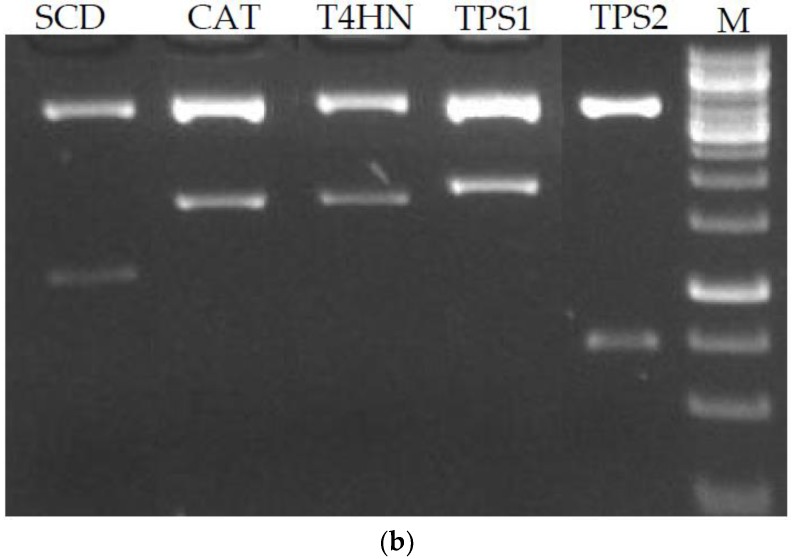
Construction of fungal vector. (**a**) Map of pM2Z-fun with zeocin resistance gene and *ToxA* promoter to express fungal genes of interest; (**b**) Restriction digestions using *Eco*RI to confirm the presence of RNAi constructs insertion. M = 1-kb DNA ladder.

The MCS and zeocin cassettes (synthesized and confirmed by sequencing) were cloned into the pMZ vector to construct the pM2Z-fun vector for expression driven by the *ToxA* promoter. The final construct is about 3.7-kb including the *ToxA* promoter, MCS, NOS terminator, and zeocin resistance gene (Figure 1a).

To test the efficiency of the new vector, five target genes from *C. protuberata* were overexpressed in the VF strain or silenced in the AN strain. Using our previous EST data, primers with specific restriction enzyme sites were designed to amplify full-length genes (Table 1). Full-length genes were amplified and inserted into pM2Z-fun vector to generate the overexpression vectors pM2Z-fun/target gene (target genes included *T4HN*, *SCD*, *TPS1*, *TPS2*, and *CAT*). All of the insertions were confirmed by sequencing.

To construct the RNAi vectors, both sense and anti-sense fragments of genes of interest were inserted into pM2Z-fun to obtain pM2Z-fun vectors expressing target genes or antisense target genes including *T4HN*, *SCD*, *TPS1*, *TPS2*, or *CAT*. Digestion of each vector with *Eco*RI produced two fragments: a larger 3.7-kb vector fragment and a shorter 600–1100-bp sense and antisense target gene fragment (Figure 1b). These digested fragments confirmed correct insertion.

### 3.2. Molecular Analysis of Transformants

**Figure 2:**
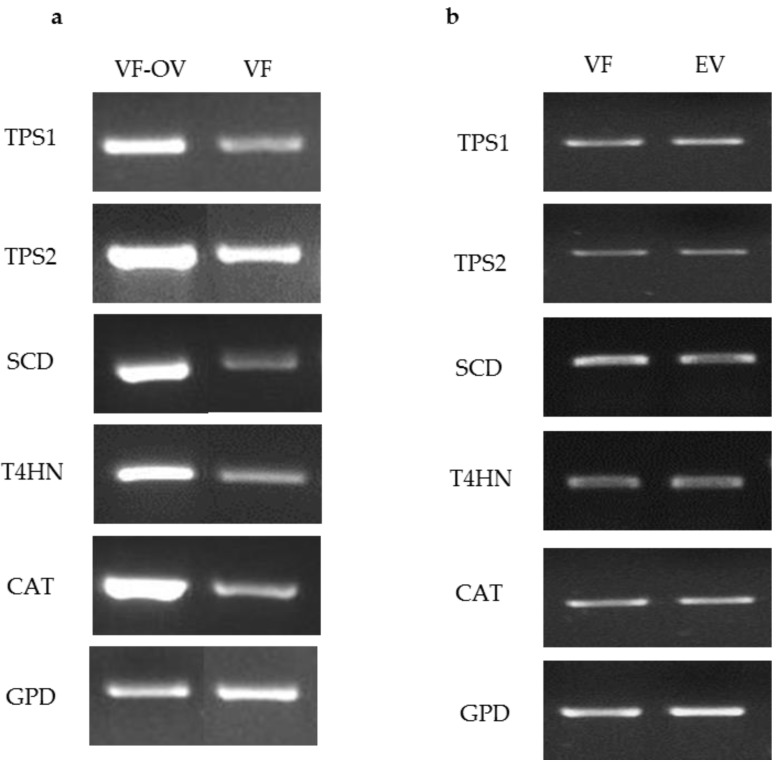
Semi quantitative RT-PCR of target genes overexpression in virus free *Curvularia protuberata* (VF transformants), VF strain and VF transformed with the empty vector. (**a**) The expression levels of target genes were higher in VF (VF-OV) than the control untransformed VF. Showing a representative RT-PCR of gyceradehyde-3-phosphate dehydrogenase (*GPD*) that was used as an internal control; (**b**) Generally, there were no changes in the expression of target genes when the VF strain was transformed with the empty vector (EV), *GPD* was also used as internal control.

**Figure 3:**
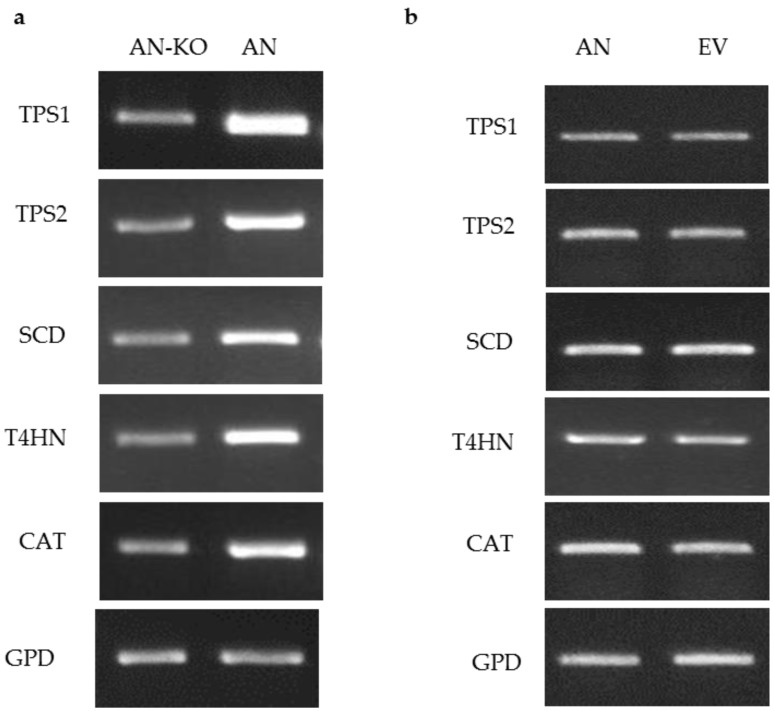
Semi-quantitative RT-PCR of target genes downregulation in *C. protuberata* AN strain (AN-KO), AN strain control and AN strain transformed with empty vector. (**a**) The expression levels of target genes in AN downregulation were lower than the control untransformed AN strain. Showing a representative RT-PCR of gyceradehyde-3-phosphate dehydrogenase (*GPD*) that was used as an internal control; (**b**) There were no changes in the expression of target genes when the AN strain was transformed with the empty vector (EV), *GPD*, again, was used as an internal control.

To test the ability of the *ToxA* promoter to drive expression of target genes, pM2Z-fun carrying *T4HN*, *SCD*, *TPS1*, *TPS2*, or *CAT* was independently introduced into the VF strain, and the transformants were designated as VF-*T4HN*, VF-*SCD*, VF-*TPS1*, VF-*TPS2*, or VF-*CAT*, respectively. Similarly, the respective RNAi vectors were individually introduced into the AN strain to generate transformants AN-*T4HN*, AN-*SCD*, AN-*TPS1*, AN-*TPS2*, or AN-*CAT*. To screen for the presence of transgenes in the transformants, 20 zeocin-resistant colonies were randomly picked to PCR for amplification with the *ToxA* forward primer and the appropriate gene-specific reverse primer. More than 80% of these zeocin-resistant colonies showed amplification products consistent with the insertion of the respective vector (data not shown). Semi-quantitative RT-PCR of target genes overexpression in virus-free *C. protuberata* (VF transformants) showed higher expression of the target genes compared to the control VF strain (Figure 2a). In addition, transformation of the VF strain with empty pM2Z-fun showed no changes in the expression of target genes (Figure 2b). The *GPD* gene that was used as an internal control showed no changes in gene expression between transformed and non-transformed strains (Figure 2a,b). On the other hand, introduction of each of the RNAi vectors into the AN strain resulted in lower expression of each of the target genes compared to expression in the wild-type AN strain (Figure 3a). Higher expression of target genes in the VF transformants was due to the ability of the *ToxA* promoter to successfully drive the expression of these heterologous genes in VF *C. protuberata*. The downregulation of each specific target gene in AN occurred due to suppression of expression by the RNAi vector. Similarly, transformation of the AN strain with empty pM2Z-fun showed no changes in the expression of target genes (Figure 3b).

### 3.3. Melanin Analysis

**Figure 4:**
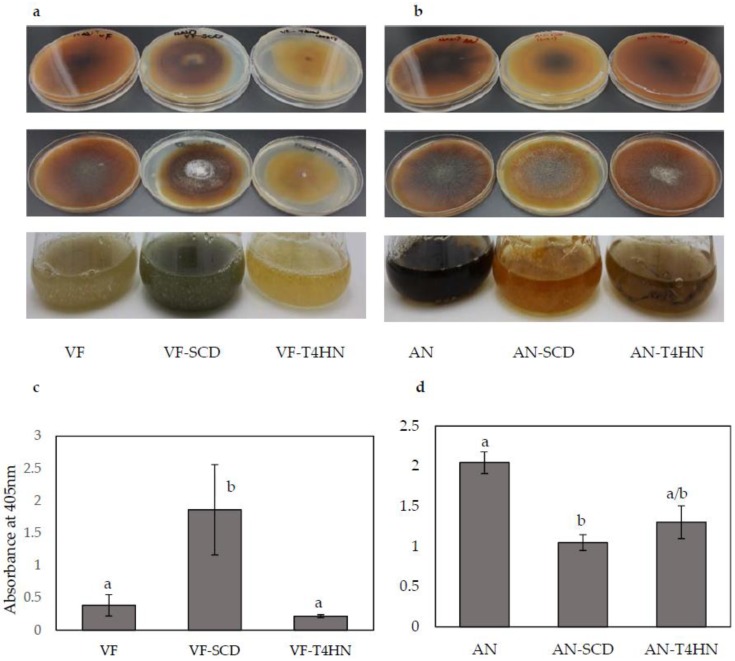
Phenotypic changes in response to melanin overexpression and downregulation and quantitative melanin analysis. (**a**,**b**) Potato Dextrose Agar plates, back (**top row**) and front (**middle row**), and liquid culture (**bottom row**); (**c**,**d**) Quantitative measurement of the melanin in control and altered strains of VF and AN. Bars represent the ±SD of three independent replications. Data were analyzed by Student’s *t*-test using Excel. Different letters above the bars indicate significance between treatments (*p* < 0.05).

After 14 days of incubation at 26 °C, the PDA plates containing VF-*SCD* (overexpression) were darker than the control VF and VF-*T4HN* (overexpression) plates (Figure 4a), which suggests higher levels of melanin were synthesized by VF-*SCD* than other strain/vector combinations. Similarly, the five-day-old liquid culture of VF-*SCD* was also darker than that of VF and VF-*T4HN*. Quantitative measurements of the melanin contents of the three strains matched the observed phenotypes: melanin content in VF-*SCD* was significantly (five-fold) higher than in the VF strain (Figure 4c). Unlike overexpression of *SCD*, overexpression of *T4HN* has no significant effect on melanin content (Figure 4c). Both AN-*SCD* and AN-*T4HN* produced yellow-brown mycelia on PDA plates and in liquid culture, while the AN strain produced dark brown mycelia (Figure 4b). The melanin concentration of the AN strain was significantly higher than those of both the AN-*T4HN* and AN-*SCD* strains (Figure 4d). Similar to overexpression data, downregulation of *T4HN* had little effect on melanin biosynthesis in *C. protuberata*.

### 3.4. Trehalose Assay

**Figure 5:**
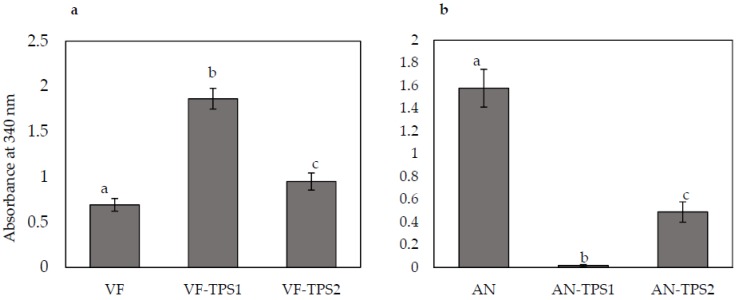
Changes in trehalose content in response to overexpression or knockdown of *TPS1* or *TPS2* expression. (**a**) VF, virus-free *Curvularia protuberata*; VF-*TPS1*, VF overexpressing *TPS1*, which encodes trehalose-6-phosphate synthase; VF-*TPS2*, VF overexpressing *TPS2*, which encodes trehalose-6-phosphate phosphatase; (**b**) AN, *C. protuberata* carrying the *Curvularia* thermotolerance virus; AN-*TPS1*, *TPS1* expression knockdown in AN; AN-*TPS2*, *TPS2* expression knockdown in AN. Bars represent the ±SD of three independent replications. Data were analyzed by Student’s *t*-test using Excel. Different letters above the bars indicate significance between treatments (*p* < 0.05).

To investigate whether *TPS1* and *TPS2* overexpression and downregulation strains were associated with any changes in trehalose content, we assayed control and transformed strains for trehalose content. Nicotinamide-adenine dinucleotide phosphate (NADPH), a product of trehalose breakdown, can be measured as an increase in absorbance at 340 nm. Overexpression of the *TPS1* and *TPS2* genes in the VF strain significantly increased trehalose accumulation. *TPS1* overexpression doubled trehalose content and *TPS2* overexpression increased trehalose accumulation by 30% compared to the control VF strain (Figure 5a). AN strains with downregulated expression of *TPS1* and *TPS2* showed significantly decreased trehalose accumulation compared to the control AN strain (Figure 5b). Downregulation of *TPS1* expression had a greater effect on trehalose accumulation than did *TPS2* downregulation.

### 3.5. Catalase Assay

**Figure 6:**
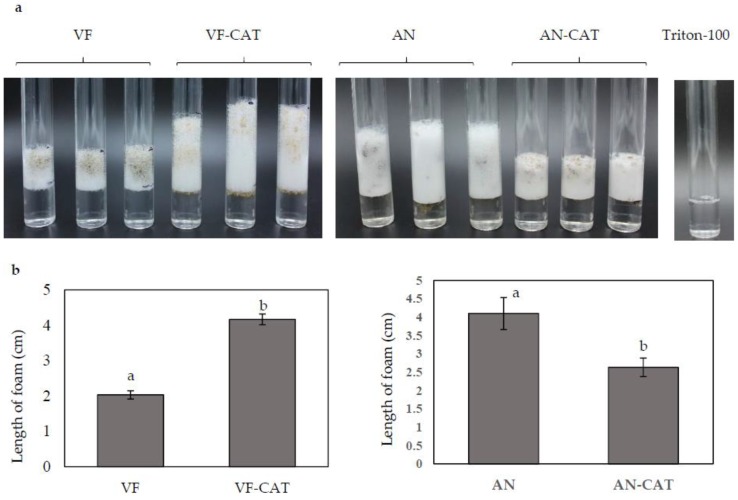
Catalase activity in *C. protuberata* with *CAT* gene overexpression in the VF strain or *CAT* downregulation in the AN strain. (**a**) The catalase activity of each fungal strain/vector combination was determined as the depth of the column of O_2_ bubbles formed; (**b**) The average column depth for three replications is presented. No bubbles formed in the control with only H_2_O_2_ and Triton-100. Bars represent the ±SD of three independent replications. Data were analyzed by Student’s *t*-test using Excel. Different letters above the bars indicate significance between treatments (*p* < 0.05).

Catalase activity was assayed in the overexpression and downregulation strains carrying the *CAT* gene by measuring the trapped O_2_ generated upon reaction of catalase with H_2_O_2_. Stable trapped oxygen was measured as the depth of bubbles formed 5 min after starting the reaction. The VF-*CAT* strain formed an average of 4.2 cm O_2_ foam compared to an average of 2 cm foam in the VF strain (Figure 6a,b). The Tritron-100 with H_2_O_2_ control without catalase showed no O_2_ production. This data suggested that overexpression of *CAT* directly increased catalase activity in *C. protuberata*. When the H_2_O_2_ plus Triton-100 was added to the AN or AN-*CAT* strains, the AN-*CAT* strains generated about 35% less O_2_ gas than did the AN strain (Figure 6a,b). These results indicate that catalase/peroxidase activity in AN-*CAT* was lower than that in the AN strain. Downregulation of *CAT* decreased catalase/peroxidase activity in *C. protuberata*.

## 4. Discussion

A new fungal expression vector (pM2Z-fun) was generated for the expression of genes of interest under control of the *ToxA* promoter with zeocin resistance as a selectable marker for transformed fungi. This simple expression vector successfully expressed targeted genes in *C. protuberata*. The pM2Z-fun vector functions well in both overexpression and RNAi lines. Semi-quantitative RT-PCR confirmed that the targeted genes were expressed at higher levels in all of the overexpression VF strains than in the control VF strain. In most fungal gene studies, knockouts are the main method that is used to analyze gene function [43,44]. Instead of knocking out the target genes, we used RNAi technology to downregulate the expression of specific target genes. Semi-quantitative RT-PCR results showed that the expression of the targeted genes was indeed reduced by introducing the RNAi vector carrying *TPS1*, *TPS2*, *SCD*, *T4HN*, or *CAT* into AN strain. Some metabolites in *C. protuberata*—such as melanin, trehalose, and catalase—may have important functions in fungal thermotolerance [2]. The biosynthesis of these compounds was affected by changing the expression of target genes affecting their synthesis in *C. protuberata*.

The overexpression of the *SCD* gene in VF-*SCD* and the *T4HN* gene in VF-*T4HN* was higher than in VF. Unlike the expression of *SCD*, which yielded a significant increase in melanin synthesis, the overexpression of *T4HN* resulted in slightly reduced synthesis of melanin. However, downregulation of either *SCD* or *T4HN* caused significant decreases in melanin accumulation. In the melanin synthesis pathway, 1,3,6,8-tetrahydroxynaphthalene reductase reduces 1,3,6,8-tetrahydroxynaphthalene to scytalone, and *SCD* catalyzes the dehydration of scytalone to 1,3,8-trihydroxynaphthalene (*T3HN*) and vermelone to dihydroxynaphthalene (*D2HN*) [45,46,47]. However, *T4HN* can also be oxidized to flaviolin [46]. In AN-*T4HN*, melanin concentration was low because of the lack of *T4HN* reductase, which could result in accumulation of flaviolin instead of scytalone. It seems that overexpression of the *T4HN* reductase gene had a negative effect on melanin synthesis in *C. protuberata*. Extra *T4HN* reductase might result in accumulation of scytalone, which would require increased *SCD* dehydratase activity to dehydrate scytalone to *T3HN*. Limitation of the *SCD* dehydratase activity in fungus would mean that less vermelone could be dehydrated to *D2HN*, which would then be oxidized to melanin. In VF-*T4HN*, lower accumulation of melanin might be caused by a lack of *SCD* scytalone dehydratase to produce sufficient *D2HN*.

Fungal trehalose biosynthesis is catalyzed by *TPS1* and *TPS2*, two main enzymes in the trehalose synthase complex [48]. The *TPS1* subunit catalyzes the formation of trehalose 6-phosphate (T6P), which is then dephosphorylated to trehalose by the *TPS2* subunit [49]. We found that overexpression of *TPS1* and *TPS2* resulted in increased accumulation of trehalose in *C. protuberata*, while downregulation of *TPS1* and *TPS2* expression diminished trehalose accumulation. Similar results have been reported in yeast and other fungi [30,50,51]. Furthermore, T6P mediates *TPS1* to regulate sugar influx which can relate to trehalose synthesis. Up- or downregulation of the *TPS1* gene might directly cause increases or decreases of the abundance of T6P as a substrate for *TPS2* to synthesize trehalose. However, downregulation of expression of the *TPS2* gene leads to the accumulation of T6P instead of trehalose.

H_2_O_2_ generated within cells could be detoxified by *CAT* or other enzymes. H_2_O_2_ can permeate cells directly; therefore, a reaction between H_2_O_2_ and catalase can be observed immediately upon addition of H_2_O_2_ to fungus. In the catalase assay, the depth of O_2_ foam indicated the relative activity of catalase in each fungal strain/*CAT* combination. Overexpression of the *CAT* gene in the VF strain resulted in twice the catalase activity of the wild-type VF fungus. On the other hand, downregulation of *CAT* gene expression in the AN strain leads to lower catalase activity. Similar results have been observed in *Magnaporthe oryzae*, where disruption of the *CAT* gene (*CPXB* in *M. oryzae*) significantly diminishes catalase activity [52], which is subject to transcriptional control.

In summary, we have generated a simple expression vector, pM2Z-fun, from which expression of a cloned gene is driven by the *ToxA* promoter. We showed that this newly synthesized expression vector could be used to overexpress or downregulate five *C. protuberata* genes that might be involved in the control of the plant, fungus, and virus three-way symbiosis. pM2Z-fun could also be useful for molecular genetic studies in other Ascomycota fungi.

## Figures and Tables

**Table 1:** Primer sequences used to clone target genes for overexpression and downregulation, and for semi-quantitative RT-PCR. Underlined sequence showing the restriction enzyme sites.

Gene	Primers	Sequence 5′→3′	Restriction Enzyme
Overexpression			
*TPS1*	Forward	TCGAATTCATGCCTGACGAACCCACAAGAC	*Eco*R1
	Reverse	GAGGATCCTCATTGGGCATTGGCAGGAGCAG	*Bam*H1
*TPS2*	Forward	GTGAATTCATGAGTGCCCCTACCGATGACAAG	*Eco*R1
	Reverse	TGCAGTCTAGACTATGGCACCGCCCGAGACTCAG	*Xba*I
*SCD*	Forward	CAGAATTCATGTTTGAGAAGAACAAACTCC	*Eco*RI
	Reverse	CACTGCAGTTACATGGCCAGCCCTGGCGCCTTC	*Pst*I
*T4HN*	Forward	TTGAATTCATGGTCATCAACGTTCCCAC	*Eco*RI
	Reverse	TCGGATCCCTACTGGGATGATCCACCAGAG	*Bam*HI
*CAT*	Forward	CAGAATTCATGTCCAAAGGCGAGTGTCC	*Eco*RI
	Reverse	CTGGATCCTCAAGTCGACTTGTTCTTGAC	*Bam*HI
Downregulation			
*TPS1*	Forward Sense	CAGCAAGCTTGAATTCGCTCCGAGATCTACCGAATC	*Eco*RI/*Hind*III
	Reverse Sense	CAAACGGATCCGTGGAAGAAACAAGGCAGACG	*Bam*HI
	Forward Anti-sense	TCCACGGATCCAAACTTACCATTGATGCGGCC	*Bam*HI
*TPS2*	Forward Sense	CACCAAGCTTGAATTCACCTATCCCCGTTGATCCCA	*Eco*RI/*Hind*III
	Reverse Sense	ACGTGGATCCACAATGTCGCCTGGCTTGTA	*Bam*HI
	Forward Anti-sense	TTGTGGATCCTCCGTCGGCAGGCTCATTTTG	*Bam*HI
*SCD*	Forward Sense	CACCAAGCTTGAATTCAGCTACGACAGCAAGGACTG	*Eco*R1/*Hind*III
	Reverse Sense	GCTACTGCAGTCCACTCGCCGTCAATCTTC	*Pst*I
	Forward Anti-sense	GCACCTGCAGACGCATCCGTGTATCGCTG	*Pst*I
*T4HN*	Forward Sense	GACTAAGCTTGAATTCAGCCAACGAAGTGTGCGAC	*Eco*RI/*Hind*III
	Reverse Sense	TCAAGGATCCTGGCTCGCCATAAGCGACTCG	*Bam*HI
	Forward Anti-sense	AGCCAGGATCCTTGATGCCACCGGGGGCGAC	*Bam*HI
*CAT*	Forward Sense	CTTCTCTAGAGAATTCGCGCTTTGCTCCTCTCAATG	*Eco*RI/*Xba*I
	Reverse Sense	GGAAAGGATCCTGGCAAGGTCCTCTGAGTTG	*Bam*HI
	Forward Anti-sense	GCCAGGATCCTTTCCATATCGTTCATAGCC	*Bam*HI
Semi-quantitative RT-PCR			
*TPS1*	Forward	TGACGAACCCACAAGACTGG	
	Reverse	CTCCTCCCGCAGCATAGAAG	
*TPS2*	Forward	GACATTGGCCTCATTACCAG	
	Reverse	CTTCGTTTTGCCAGCTCAT	
*SCD*	Forward	AACTCCAGCCTACCTTTGAGG	
	Reverse	ACTCGTACCACCGAATGTCC	
*T4HN*	Forward	CACCATGGTCATCAACGTTCCCA	
	Reverse	TACTTCTCCTCGCTAATCTCC	
*CAT*	Forward	GTGCCTGGTTCAAGCTTCTC	
	Reverse	TGAACGTCAGTCTGCTCCTG	
*GPD*	Forward	GCAACAACCTGACCGTCAAC	
	Reverse	CCCACTCGTTGTCGTACCAA	
